# Prognostic Significance of Melanoma Differentiation and Trans-Differentiation

**DOI:** 10.3390/cancers2020989

**Published:** 2010-05-26

**Authors:** Nityanand Maddodi, Vijayasaradhi Setaluri

**Affiliations:** Department of Dermatology, University of Wisconsin School of Medicine and Public Health, 1300 University Avenue, B25, Madison WI 53706, USA; E-Mail: nmaddodi@dermatology.wisc.edu

**Keywords:** melanoma, melanocyte, prognostic markers, differentiation, trans-differentiation

## Abstract

Cutaneous malignant melanomas share a number of molecular attributes such as limitless replicative potential that define capabilities acquired by most malignancies. Accordingly, much effort has been focused on evaluating and validating protein markers related to these capabilities to function as melanoma prognostic markers. However, a few studies have also highlighted the prognostic value of markers that define melanocytic differentiation and the plasticity of melanoma cells to trans-differentiate along several other cellular pathways. Here, we provide a comprehensive review and evaluation of the prognostic significance of melanocyte-lineage markers such as MITF and melanogenic proteins, as well as markers of vascular epithelial and neuronal differentiation.

## 1. Introduction

The incidence of cutaneous malignant melanoma, the deadliest skin cancer, is increasing rapidly. In the United States alone, it is estimated that in the year 2009, about 68,720 persons were diagnosed with melanoma, resulting in 8,650 deaths [1], and representing 8,780 more persons being diagnosed and 540 more deaths due to melanoma in the year 2009 compared to the year 2007. Since the majority of the newly diagnosed cases of cutaneous melanoma are early stage lesions localized in the skin, surgical resection of the lesion often provides effective cure. The widely used evidence-based current system of clinical staging, which is known as tumor-node-metastasis (TNM) classification, was developed based on analysis of 17,600 melanoma patients and was revised recently to incorporate improved understanding of the disease and analysis of additional patients [2,3]. According to this system, tumor thickness (Breslow thickness) and ulceration are the most powerful predictors of survival in patients with localized stage I and II melanomas. However, aggressiveness of the lesion at the time of diagnosis cannot always be ascertained by these two parameters, resulting in recurrence in some individuals diagnosed with early stage lesions [4]. For example, estimates of long-term survival for patients with thin (≤1 mm) primary cutaneous melanomas vary widely. Although ulceration is a reproducible histopathological feature that worsens the prognosis of patients with equivalent thickness, in thicker melanomas, the level of invasion appears to be a better predictor than tumor ulceration in thin melanomas [2]. In the revised staging system, the level of invasion is replaced with mitotic rate for subgrouping stage I melanoma [3]. Similarly, the prognosis associated with stage III melanoma is variable and influenced more by the number of lymph nodes involved than the thickness of the primary tumor, illustrating the need for identification and validation of biological markers that can accurately and reliably predict aggressiveness of cutaneous melanoma. Tissue biomarkers are useful and essential tools in the diagnosis and prognostic classification of cancer and also serve as valuable measures in monitoring the clinical course of the disease and its response to therapy [5]. Biomarkers represents measureable quantitative changes in the diagnostic indicator that depict the risk or presence of the disease [6]. In this review, we summarize and critically examine the published literature on genes and proteins related to melanoma differentiation and trans-differentiation as prognostic markers and potential targets for therapy.

## 2. Melanoma Biomarkers Based on Shared Common Features of Cancers

Cancer can be viewed as a manifestation of alteration of six essential features and stress phenotypes of eukaryotic cells that collectively initiate and maintain malignant growth [7,8]. These alterations culminate in self-sufficiency in growth signals, insensitivity to growth-inhibitory signals, evasion of programmed cell death (apoptosis), limitless replicative potential, various forms of cellular stress, sustained angiogenesis, and tissue invasion and metastasis [7,8]. Any single alteration alone or in combination with other alterations could therefore potentially serve as useful prognostic indicator(s) and/or target(s) for cancer therapy. The value of these altered cellular features for prognosis of cutaneous melanoma has been extensively investigated and critically evaluated in a recent and excellent review [9]. In addition to the six acquired capabilities of cancer, altered cellular differentiation is another well recognized feature of cancers [10,11]. It is generally believed that “malignancy results from genetic changes that uncouple the normal balance between multiplication and differentiation” [10]. Consistent with this notion, cancer cells often exhibit characteristics of dedifferentiation. A corollary of this phenomenon is that tumor cell differentiation/trans-differentiation and expression of specific differentiation-related proteins could serve as prognostic markers. 

## 3. Melanocyte Differentiation Proteins as Melanoma Biomarkers

The hallmark of differentiated melanocytes, which are derived from the neural crest, is the presence of melanin pigment. Micropthalmia associated transcription factor (MITF) is the critical master regulator of melanocyte development, including commitment and survival of melanoblasts and their differentiation along the melanocytic lineage. MITF regulates several melanocyte-specific genes involved in melanogenesis (reviewed in [12,13]). Therefore, high levels of MITF expression in primary melanoma could confer either a survival advantage or potential for terminal melanocytic differentiation. However, MITF expression has been associated with both poor prognosis as well as better prognosis of melanoma, depending on the stage of the tumor. In 63 patients diagnosed with intermediate-thickness melanoma, using univariate analysis of MITF expression, Salti *et al.* reported that patients with tumors expressing higher levels of MITF showed a better mean overall survival (OS) and disease-free survival (DFS) [14]. Although this study showed statistically significant increases in OS and DFS (P = 0.0086 and P = 0.0054, respectively) and in both univariate and multivariate analysis, since this study lacked data on confidence intervals, it did not qualify for inclusion in the high quality cohort studies on melanoma biomarkers [9].

A high percentage of metastatic tumors and a subset of primary melanomas have been found to harbor amplification of the MITF gene. Such amplification and consequent higher levels of MITF protein expression in metastatic melanoma was reported to be associated with a decrease in five-year survival of patients with metastatic disease [15]. This study also did not qualify for inclusion as a high quality cohort as described by Gould Rothberg *et al*. [9]. Thus, the prognostic significance of MITF gene amplification and its expression levels to cutaneous melanoma progression needs further validation.

Melanocortin 1 receptor (MC1R) is a G-protein coupled receptor and a transmembrane protein specifically expressed in melanocytes, which plays an essential role in stimulation of melanogenesis by binding to alpha-MSH in keratinocytes and melanocytes in the skin [16]. More than 70 allelic variations have been identified in *MC1R*. Polymorphisms in the *MC1R* gene are known to be associated with the diversity of human pigmentation [17]. Melanoma risk is associated in the individuals with the pale skin pigment and individuals with selected *MC1R* gene variants have an increased risk for melanoma development that is independent of skin type and hair color [18,19,20]. However, to our knowledge, the prognostic value of *MC1R* variants or their expression levels in primary cutaneous melanoma has not been examined. 

Tyrosinase is the key and limiting enzyme in melanin biosynthesis, and hence a definitive marker of melanocyte differentiation. Prognostic value of detection of tyrosinase-positive circulating tumor cells, as detected by RT-PCR, has been studied extensively [21,22,23,24,25,26]. But there are only a few studies on the prognostic significance of expression of tyrosinase or its levels in the primary or metastatic lesion *per se* [27]. Tyrosinase-related protein 1 (TYRP1) is a melanocyte-specific gene product involved in the biochemical modulation of melanin formation. In a study aimed to search for new molecular markers associated with melanoma progression, Bolander *et al*. found expression of TYRP1 to be inversely correlated with tumor stage but not associated with overall or disease-free survival, suggesting no correlation between TYRP1 expression and survival [28]. Tyrosinase-related protein 2 (TYRP2) is a melanocyte-specific enzyme that is involved in the melanin biosynthetic pathway and also expressed in other neuroctodermal malignancies such as retinoblastomas [29], gliomas [30], and glioblastomas [31]. Takeuchi *et al*. studied mRNA expression of TYRP1 and TYRP2 in stage IV melanoma patients’ lesions and concluded that elevated levels of TYRP1 and TYRP2 mRNA correlated with improved overall survival [32]. Other melanogenic proteins that were evaluated for melanoma prognosis include melanocyte-specific immunological target proteins gp100 and melanA/MART1. Hofbauer *et al.* reported that loss of gp100 and tyrosinase expression in primary melanoma showed a negative survival trend when compared to their uniform expression. Studies on prognostic value of melanA/MART1 expression, however, have produced contradictory results [27,33,34]. Interestingly, while melanogenic proteins may still hold some prognostic value, pigmentation, the most remarkable differentiation-related feature of melanocytes, has been shown very early on to be of no prognostic significance [35].

Melastatin/TRPM1 is a member of a transient receptor potential (TRP) family of ion-channel proteins, which are being increasingly implicated in cancer [36]. TRPM1 is a calcium-channel protein that is selectively expressed in melanocytes in the skin and the eye. TRPM1 expression in melanocytes appears to correlate with differentiated functions, including melanin pigmentation [37,38]. TRPM1 was originally identified as a marker associated with aggressiveness of murine melanoma and growth inhibition in human melanoma cell lines [39,40]. Using multivariate Cox regression analysis, Duncan *et al.* showed that uniform TRPM1 mRNA expression in the primary tumor was correlated with prolonged disease-free survival [41]. Similarly, Hammock *et al.* also showed that increasing loss of TRPM1 mRNA in primary melanoma correlated with aggressive metastatic melanoma [42]. Both these studies used *in situ* hybridization analyses. However, since TRPM1 produces multiple transcripts by alternative splicing [40] and expression of the shorter isoforms are reported to regulate the function of the full-length isoform [43], immunohistochemical evaluation of protein isoform expression may be necessary for validation of TRPM1 as a prognostic marker.

## 4. Prognostic Value of Melanoma Trans-Differentiation

Tumor trans-differentiation is a phenomenon where tumor cells change their fate and differentiate into other cell types. Although cutaneous melanomas tend to maintain certain characteristics of melanocytic differentiation, they also exhibit plasticity to differentiate along multiple cellular pathways including endothelial and neuronal pathways [44]. 

### 4.1. Vasculogenic Mimicry

The plasticity of tumor cells to trans-differentiate into endothelial cells and their ability to give rise to fluid-conducting matrix-rich mesh capable of mimicking the circulatory system, termed as vasculogenic mimicry (VM), has been described [45,46,47,48,49,50,51,52,53]. Development of vasculogenic-like networks and matrix remodeling were observed in aggressive melanomas [54]. Analysis of microdissected vascular-like network showed that expression of angiogenesis-specific genes were differentially regulated among the phenotyically distinct melanoma structures in VM. Adjacent nest-cells overexpress extracellular matrix-related genes, suggesting melanoma plasticity could enable autopoiesis of VM elements within the tumor infrastructure [55]. Thies *et al*. studied the prognostic significance of induction of VM in cutaneous malignant melanoma and correlated melanoma metastasis in a 10-year follow-up study and concluded that presence of PAS-positive loops and networks were highly significant prognostic markers of metastasis in cutaneous melanoma [56]. Another study showed a significant decrease in disease-free survival among patients whose tumors contained cross-linked and networking patterns [57]. These studies point to a strong association between VM and poor outcome [46]. However, this conclusion was questioned by another study that showed no significant difference in distribution of PAS-positive patterns between cases and control, suggesting no prognostic role of VM in thick cutaneous melanomas [58]. 

### 4.2. Neural Differentiation

Expression of neural specific proteins in melanoma has been described long ago and is consistent with the tendency of neoplastic melanocytes to differentiate along the neural pathway [59,60,61]. For example, invasive primary melanoma cells adopt a phenotype that includes the expression of certain proteins typical to neurons. Microtubule associated protein 2 (MAP2) is more frequently and abundantly expressed in melanocytic nevi and early primary melanoma than in the metastatic melanoma lesion [61,62]. MAPfamily proteins bind to and stabilize microtubules. MAP2 is neuronal specific marker, generally localized to the dendrites of postmitotic terminally differentiated neurons. MAP2 is expressed abundantly in early invasive primary melanoma lesions, but not found in metastatic melanoma lesion and cell lines. Immunohistochemical staining for MAP2 expression and survival analysis of a small cohort of patients diagnosed with malignant melanoma showed that MAP2 expression is inversely related to tumor aggressiveness and that MAP2 is a significant prognostic indicator of disease-free survival in patients diagnosed with primary melanoma [62]. In a meta-analysis of 1797 published reports, the study on MAP2 as prognostic marker was validated as a high quality cohort study that included and reported several critical criteria such as multivariate analysis, hazard ratio and confidence intervals of data [9]. 

Ectopic expression of MAP2 in metastatic melanoma cells lines leads to microtubule stabilization, cell cycle arrest in G2-M phase and growth inhibition of metastatic melanoma cells *in vitro* [62]. Recently Gambichler *et al*., studied MAP2 expression in melanocytic lesions and showed that MAP2 expression in dysplastic nevi and superficial spreading melanomas (SSM) was significantly increased compared to benign nevi and subcutaneous melanoma metastases. However, MAP2 expression in SSM appears to be a moderately positive predictor of aggressiveness [63]. The biological mechanism for this finding remains to be investigated. 

Induction of MAP2 in melanoma can be exploited as strategy for melanoma prevention and therapy. Studies on regulation of *MAP2* promoter in melanoma showed that *MAP2* is regulated by Notch1 signaling and by neuronal repressor HES1 and activator NeuroD [64]. Activation of Notch1 signaling induces tumor cell survival *in vitro* and enhances aggressiveness of vertical growth phase primary melanoma to metastasize *in vivo* [65]. MAP2 expression in melanoma may also be related to the activation levels of BRAF-MEK signaling. The regulatory sequences of MAP2 are progressively methylated during melanoma progression, suggesting MAP2 expression is silenced by epigenetic mechanism in metastatic melanoma [66] suggesting that treatment with demethylating agents such as 5’-azacytidine can be useful for melanoma, if used in appropriate combination with other agents.

Neural precursor cell expressed, developmentally down-regulated 9 (NEDD9) is another neuron-related marker expressed in melanoma. In the process of identification of the genomic events associated with the acquisition of metastatic potential, Kim *et al*., reported amplification of *NEDD9*. NEED9 is more frequently overexpressed in metastatic melanoma than in primary melanoma and overexpression is correlated with melanoma progression. NEDD9 enhances the invasion and metastatic potential in both melanocytes and melanoma. Based on immunochemical staining of human melanoma tumor specimens, it was suggested that NEDD9 overexpression correlates with metastatic progression in human melanomas [67]. 

Similarly, nestin, a neuroepthithelial stem cell marker, is also expressed in greater percentage of melanomas than to benign nevi. There was a significant difference in the expression pattern of nestin in primary and metastatic melanoma [68,69]. Expression of nestin significantly increased in melanoma compared with nevi and correlates with more advanced stage of the disease [70]. Nestin expression in stage I and II melanoma patients significantly predict poor survival [71,72]. Thus, the neural crest origin of melanocytes seems to be relevant for melanoma prognosis.

## 5. Concluding Remarks

Despite the impressive progress made in understanding of molecular mechanisms involved in melanoma tumorigenesis, management of patients diagnosed with primary cutaneous melanoma remains a challenge due to the lack of reliable prognostic markers. When diagnosed early, excision of the primary lesion and sentinel node surveillance are quite effective in disease management. However, recurrence in such patients that do not qualify for adjuvant treatment makes the identification of reliable and accurate prognostic markers an urgent and immediate priority. Based on the survey of literature and our own published data, we believe that there is a need for critical evaluation of differentiation and trans-differentiation markers as prognostic markers. A major challenge in biological validation of melanoma biomarkers is the lack of understanding of how the function(s) of a given marker specifically influences the behavior of melanoma cells. Therefore, it is imperative that future studies on the differentiation related markers focus on their biological roles in altering the behavior of melanoma cells. Accordingly, markers that define a post-mitotic state of terminal differentiation, as exemplified by MAP2, deserve further attention. Detailed studies on such markers have the potential to also uncover novel pathways and additional prognostic markers. Some of these proteins, such as melanocytic transcriptional factor MITF and neuronal repressor HES1, could also serve as targets for therapy. 
